# Abstract of the Proceedings of the Buffalo Medical Association

**Published:** 1862-10

**Authors:** Julius F. Miner

**Affiliations:** Secretary


ART. II.—*Abstract of the Proceedings of the Buffalo Medical Association.*

TUESDAY EVENING, September 2, 1862.

Dr. H. M. Congar, Vice President, in the Chair.

*Dr. Miner* presented for examination an arterial tumor, which had been described, as an aneurism by anastomosis, which he had that that morning removed, from the head of a child seven months old.—At birth it was observed a very small pimple, hardly perceptible, situated upon the right side of the frontal bone, near the union with the parietal. It had continued to increase in size, recently growing rapidly, until now, before removal, it measured two inches long by one wide, and was about an inch in thickness, standing up above the surrounding surface. The arteries leading to it were greatly distended and quite numerous, entering it on all sides, and giving distinct pulsation not only over their couse, but to the whole tumor. The vessels over that side of the face and scalp were also greatly distended, and when the child cried, or made violent effort of any kind, the increase of venous blood gave a dark livid color to the integument for considerable distance from the tumor. After having brought the child under the influence of sulphuric ether, incision was made about two lines from the tumor, in healthy tissue, extending only so far as to be able to control the hemorrhage by pressure until the vessels were ligated leading to the tumor. The vessels leading from it bled as freely as those leading to it, and this was restrained by grasping the edge as firmly as possible, and holding it until the tumor was surrounded and the supply vessels ligated. The tumor was removed with a loss of not more than two and a half or three ounces of blood. The child was at first faint and depressed from loss of blood and the effects of the ether, but soon rallied and seemed to suffer but little from the operation.

The various plans of operation proposed and practiced by surgeons for removal of these growths were mentioned, and the advantages and disadvantages of each plan briefly reviewed. Strangulation, starvation, compression, injection, excision and amputation, were the general heads under which might be grouped a variety of operations, all more or less efficient in the removal, and all obnoxious to some greater or less serious objections. Injection with persul ferri or perchlor ferri would, in his opinion, be found one of the operations or plans of removal most worthy of trial in cases where excision could not safely be performed, and yet the great objection would seem to be the liability of hemorrhage upon the separation of the slough, an objection which holds in almost all plans of procedure, seaton, heated needles, ligature, escharotics, or whatever other means might be suggested. Again the *pain* attending any plan of gradually removing such growths was too great for the endurance of a child of seven months. Excision when it can be performed safely was regarded as beyond all comparison, superior to any other plan yet proposed; less painful, more certain, less liable to be attended by secondary hemorrhage, producing less shock or depression, less liable to be attended by, or to produce diarrhœa, which he regarded as the most frequent and common cause of depression after all operations upon very young infants.

The tumor removed was found upon examination to consist of enlarged vessels passing through the base of the tumor, while over this layer very few vessels could be seen under the microscope. The mass of the tumor was cellular structure; the cells appearing to communicate with each other. The skin covering it was very thin, and rested upon areola tissue without any intervening structure. The cells were filled with blood, the walls of the cells thin and almost indistinct in some places, leading one to suppose he had only blood corpuscles under examination. The tumor was presented not on account of the rarity of such growths or peculiarity in its character, but to draw attention to the reasons for adopting the excising mode of operation, rather than any of the more recently suggested plans of procedure. Other cases might require, and more properly admit of different mode of removal, but tumors of this character which can safely be removed by excision can perhaps be treated in no other manner with greater satisfaction and success.

*Dr. Gay* was satisfied of one fallacy in surgical teaching. Often read and heard of the impropriety of using the suture in wounds of the scalp. Had been in the habit of drawing together wounds of scalp with suture, not removing the hair, but approximating the integument as nearly as possible, and has never seen any erysipelas or other unpleasant effects.

*Dr. Ring* had dressed hundreds of wounds of the scalp with suture, and had never seen any unpleasant effects. Thought shaving the scalp would be as likely to produce erysipelas as a suture. A very neat way of dressing wounds of the scalp was to lay the hair over each way like the lappings of the many tailed bandage. Had seen tumors like the one presented by Dr. Miner removed in New York, by heated needles, but did not know the results.

*Dr. Kempson* remarked that in the district of his former practice, wounds of the scalp were exceedingly common, the chances of such injury with the miners were as great as of being wounded in a battle. Erysipelas was not as common there as here; rare to see erysipelas in that country from injury of the scalp.

*Dr. Gay* spoke of the differences in actæa racemosa and cimicifuga racemosa, and their frequently being spoken of, as one and the same plant. Would some time prepare a paper upon the distinctions.

*Dr. Kempson* related a case of uterine hemorrhage after labor. Natural labor at full time; child expelled quickly, and placenta removed, when immediately the largest amount of blood he ever saw discharged at once, came away. To subdue the hemorrhage used opium and ice. Nodules of ice passed directly into the womb was the only thing which seemed to control the bleeding. Said there was no risk in its use, that it was perfectly safe. Patient recovered rapidly. Thought she lost two gallons of blood.

*Dr. Gay* attended a lady in miscarriage who had so profuse hemorrhage that he put pieces of ice upon the mouth of the uterus. Contraction he regarded as the great remedy in post partum hemorrhage, and pressure with the hand over the uterus or compression the most efficient means to produce it. Did not think much of ice externally; had known it seem to do injury.

*Dr. Kempson* would remark that in the case reported, he also used compression; continued it as long as he could himself, and after that, had help.

*Dr. Miner* remarked that whatever was calculated to produce or excite contraction of the uterus was proper in such cases, and that any suppression not attended by contraction was only temporary and unsafe. Thought dashing cold water upon the abdomen sometimes a most useful expedient, but that great quantities of ice, or ice water, long continued might be, and often were injurious, defeating the proper objects of treatment.

Any foreign substance, as for instance the hand introduced into the cavity of the uterus would often prove most efficient in producing contraction. In alarming and severe cases this was a most ready plan, and the hand would often be grasped with great violence, so that the attendant would be glad when liberated. Ice might and probably would act in something the same way. That it would or would not have any advantages over the hand he was unable to say, but had the impression that the hand would be found to answer the same purpose. In regard to compression over the uterus with the view to control hemorrhage or produce contraction, he had no confidence in its efficacy It was unquestionably proper with the view of supporting the walls of the distended abdomen and uterus, to apply the usual compress and bandage, but to think of shutting up the uterus or preventing hemorrhage by compression of its walls, was in his opinion exceedingly absurd, and he could conceive of no benefit from severe and long continued pressure over the uterus in cases of hemorrhage.

*Dr. Ring* had had many severe cases, and should be glad to learn any thing useful in the treatment. Has found giving large doses of ergot the most efficient means before the completion of labor, and immediately after making compression. Opium he is theoretically opposed to; has had no experience in its use. Believed in cold water, and said the womb was not exactly muscle. Thought the introduction of ice the very best remedy, and that Dr. Kempson was worthy of great credit in saving his patient. In cases of severe hemorrhage we must let stimulants go, and take care of the bleeding.

*Dr. Kempson* had always been taught to make compression; to always feel for the uterus and make compression upon it; regarded it as of the greatest importance; would mould, and as it were form the uterus as he would have it. Spoke of a case where he threw open the windows, and snow being on the ground sent for snow, and introduced snow balls. The patient was almost moribund. It acted like a charm. Cold acts as a sedative, and in that way is useful; was obliged to Dr. Ring for the suggestion of giving ergot; will give ergot a trial.

*Dr. Gay* said the Secretary had asked the use of pressure over the uterus. He thought it would approximate its sides, and thus have a tendency to suppress bleeding; would support a uterus which had been overworked and debilitated. Introduction of the hand, as suggested by him, before the discharge of the after-birth might stimulate contraction, but after that it was an entirely different case. Had often removed coagula of blood with relief of all the trouble; thought compression the all sufficient remedy. Objected in toto to the introduction of the hand into the uterus after delivery, and said that no woman who had been thus served, would be likely to submit to similar treatment again.

*Dr. Miner* remarked that he was satisfied with the position, that pressure, other than the support he had described, over the unsupported uterus after delivery, could be of no service in arresting hemorrhage; while the hand introduced into the uterine cavity either before or after the delivery of placenta, was one of the most efficient, indeed the most efficient means for inducing contraction of that organ and consequent arrest of hemorrhage. While Dr. Gay objects to the introduction of the hand only as a dernier resort, he still says he has often removed coagula of blood, and thus at once been able to arrest the hemorrhage without further trouble. Now the question is, how can it be known that there is retained coagula of blood without introducing the hand? and how can it be removed without using the same means?

*Dr. Gay* replied that he could tell that coagulated blood was retained in the uterus by feeling through the abdominal walls.

*Dr. Ring* believed that pressure over the uterus made it contract; and that we shall never have hour-glass contractions if we adopt that practice.

*Dr. Congar* would endorse Dr. Miner. He was never taught to use pressure such as had been described. Had been learned to feel for the fundus of the uterus to learn if it was contracted and the patient safe from liability to hemorrhage. The only object was stimulation of contraction. Cold water poured from a height was a very useful expedient. Ice, he had used, but always kept the patient warm; also kept the head low and feet elevated.

*Dr. Kempson* thought there was no disagreement, pressure and manipulation being useful, and all agreeing in their use.

*Dr. Gay* thought we agreed sufficiently, but had perhaps misunderstood each other. Liked the familiar way of discussion.

The Secretary inquired if the Society would have the transactions published verbatim, as the discussion had been conducted?

Many of the members expressed the wish to have it so published.

Voted to adjourn to Tuesday evening, Oct. 7, 1862.

JULIUS F. MINER, Secretary.

